# Development and Psychometric Validation of an Intercultural Mediation Questionnaire for Law Enforcement Personnel in Spanish Border Regions

**DOI:** 10.3390/bs16071090

**Published:** 2026-07-02

**Authors:** José Alejandro Torres-Aranda, Christian Fernández-Leyva, María Tomé-Fernández, José Manuel Ortiz-Marcos

**Affiliations:** 1 Department of Research Methods and Diagnosis in Education, Faculty of Education and Sports Sciences, University of Granada, 52071 Melilla, Spain; joseamelilla@correo.ugr.es (J.A.T.-A.); mariatf@ugr.es (M.T.-F.); 2 Department of Research Methods and Diagnosis in Education, Faculty of Education Sciences, University of Granada, 18071 Granada, Spain; leyvacf@ugr.es

**Keywords:** intercultural mediation, psychometric assessment, public employees, validation, Content Validity Index (CVI)

## Abstract

This study aimed to develop and validate a psychometric instrument to assess intercultural mediation among public employees of security forces in Spanish border contexts (CMIEPFS). An initial pool of 42 items was created and refined through expert judgment, resulting in a final 38-item questionnaire. The instrument was administered to two independent samples (*n* = 426 for exploratory factor analysis; *n* = 512 for confirmatory factor analysis). Construct validity was examined using structural equation modeling, and reliability was assessed through Cronbach’s alpha, intraclass correlation coefficient, and Pearson correlation. Results supported a three-factor structure: (1) acceptance of intercultural environments, (2) intercultural competences in the workplace, and (3) training in intercultural mediation. Exploratory factor analysis indicated adequate sampling adequacy (KMO = 0.916) and a significant Bartlett’s test (*p* < 0.001), explaining 63.08% of the variance. Confirmatory factor analysis showed an acceptable model fit (RMSEA = 0.07). Internal consistency was high (α = 0.91), and test–retest reliability was satisfactory (ICC = 0.82; r = 0.84, *p* < 0.001). The instrument shows adequate validity and reliability for assessing intercultural mediation in public security personnel. It provides a useful tool for research, training evaluation, and institutional diagnosis in multicultural professional contexts.

## 1. Introduction

Intercultural mediation is currently conceptualized as a complex process aimed at facilitating communication, preventing conflicts, and promoting equitable relationships among culturally diverse individuals and groups, integrating cognitive, affective, and behavioral dimensions. In contemporary research, this construct is closely associated with intercultural competence, defined as the ability to interact effectively and appropriately in multicultural contexts, particularly within institutional and professional settings characterized by power asymmetries and specific organizational norms (Deardorff & Jones, 2021; Gómez et al., 2023). From this perspective, intercultural mediation extends beyond the management of explicit conflicts and includes preventive competencies such as cultural empathy, intercultural communication, and context-sensitive decision-making.

Recent studies conceptualize intercultural competence as a multidimensional and dynamic construct that can be developed through training and professional experience, thereby influencing levels of intercultural acceptance within specific contexts (Koehler & Filipps, 2024). Within this framework, mediation is understood as a set of attitudes and skills (Escribano & Martínez-Buján, 2014) that constitute a specific dimension of intercultural competence. Accordingly, similar to intercultural competence itself, mediation can be enhanced through targeted training interventions (Perlinger & Wischmann, 2025).

Furthermore, empirical evidence suggests that unidimensional models are insufficient to capture the complexity of intercultural interactions in real-world settings. Consequently, the use of instruments that integrate attitudinal, cognitive, and behavioral dimensions in a balanced manner is recommended (Genkova & Schreiber, 2025). In this context, the development of original questionnaires enables the adaptation of the construct to specific professional environments, provided that rigorous psychometric validation procedures are followed (Bekere & Teketel, 2024). Therefore, the aim of this study is to develop and validate a questionnaire designed to assess intercultural mediation competencies among law enforcement personnel working in Spanish border contexts.

Spanish border contexts are characterized by high levels of cultural diversity resulting from migration flows, cross-border mobility, and ethnic plurality. This situation places public employees working in law enforcement agencies in a key position as informal mediators between the State and citizens (Deller-Wessels et al., 2022). Recent scientific literature highlights that interactions between security personnel and culturally diverse populations constitute critical scenarios in which a lack of intercultural competence may lead to misunderstandings, institutional mistrust, and the escalation of conflicts (Kim et al., 2022). Within this framework, intercultural mediation emerges as an essential professional competence to ensure proportional, legitimate, and human rights-based interventions (Palacio & Hays, 2025).

Several studies have shown that intercultural training within law enforcement agencies contributes to improving perceptions of police legitimacy and fostering community cooperation, particularly in territories characterized by high sociocultural complexity (Dai & Feng, 2025). However, there remains a significant lack of validated instruments specifically designed to assess intercultural mediation competencies within these professional groups. This methodological gap reinforces the need to develop and validate contextually relevant questionnaires capable of reflecting the real demands of professional practice in border environments.

The development of original instruments based on existing scales is a common practice in applied research, particularly when available tools do not fully align with the target context or population (Al-Afifi et al., 2026). Recent psychometric literature emphasizes that such instruments should be grounded in a comprehensive theoretical review of the construct and in the critical integration of previously validated dimensions, avoiding the mere aggregation of items without conceptual coherence (Boateng et al., 2018). In the case of intercultural mediation, this implies selecting indicators that reflect both individual competencies and situated professional practices.

Given the absence of instruments specifically designed for law enforcement personnel in border contexts (Halpern et al., 2022), the development process incorporated expert judgment procedures to ensure the relevance, clarity, and representativeness of the proposed items (Boateng et al., 2018).

Previous research (Sofyan et al., 2026) on intercultural competence consistently supports multidimensional models and highlights the usefulness of factor analytic approaches for identifying and confirming latent dimensions of the construct (Gómez et al., 2023).

Empirical studies have also demonstrated that multidimensional factorial models provide a better fit than unidimensional solutions in scales measuring intercultural competence and sensitivity, particularly in professional populations (Jin et al., 2026; Chen & Gabrenya, 2021; Genkova & Schreiber, 2025). In this regard, the validation of an intercultural mediation questionnaire in law enforcement contexts requires confirming that the identified dimensions—acceptance of intercultural environments, intercultural competencies in the workplace, and training in mediation and intercultural diversity—remain empirically distinct while being theoretically related (Mia et al., 2025).

Previous studies (Boateng et al., 2018) have shown that intercultural training programs can contribute to the development of differentiated competence profiles among professionals working in multicultural environments and can improve their ability to manage intercultural interactions effectively (Ertay & Gilanlioglu, 2024; Kazykhankyzy & Alagozlu, 2019). Consequently, the availability of psychometrically sound instruments is essential for evaluating these competencies and for assessing the effectiveness of training initiatives in law enforcement contexts characterized by cultural diversity (Mardones et al., 2024; Peifer et al., 2023).

Overall, the validation of a questionnaire designed to measure training in intercultural mediation among public employees in law enforcement agencies operating in border contexts has significant scientific and practical implications. From a research perspective, it contributes to addressing a gap in the empirical literature on the measurement of intercultural competencies in specific institutional populations, advancing knowledge on the structure and operationalization of the construct (Genkova & Schreiber, 2025). From a practical standpoint, having a valid and reliable instrument facilitates the design of evidence-based training programs and the objective evaluation of key professional competencies. Moreover, several authors agree that the rigorous assessment of intercultural mediation—particularly its training dimension—can support the development of public policies aimed at improving social cohesion, preventing conflicts, and strengthening institutional legitimacy in multicultural contexts (Sercu, 2022). In this sense, the validation of the proposed questionnaire aligns with current recommendations in applied research and responds to emerging social and professional needs in Spanish border contexts.

### Proposed Dimensional Structure of the Instrument

Based on established models of intercultural competence and mediation, a three-dimensional structure was expected prior to factor analysis. Intercultural competence has consistently been conceptualized as a multidimensional construct that integrates attitudinal, behavioral, and developmental components (Escribano & Martínez-Buján, 2014; Gómez et al., 2023; Genkova & Schreiber, 2025). Likewise, recent research has emphasized that effective intercultural mediation requires not only positive attitudes toward cultural diversity, but also the practical skills and training necessary to manage intercultural interactions in professional settings (Perlinger & Wischmann, 2025; Koehler & Filipps, 2024).

The first dimension, acceptance of intercultural environments, corresponds to the attitudinal component of intercultural competence. It reflects openness toward cultural diversity, respect for different beliefs and values, and the positive evaluation of intercultural interaction. Previous studies have identified these attitudes as a fundamental prerequisite for effective intercultural engagement and mediation (Escribano & Martínez-Buján, 2014; Gómez et al., 2023).

The second dimension, intercultural competences in the workplace, represents the behavioral and professional component of the construct. It includes empathy, communication skills, adaptability, and the ability to interact appropriately with culturally diverse populations in occupational contexts. These competences have been recognized as essential for professionals working in multicultural environments, particularly in institutional settings characterized by frequent intercultural encounters (Koehler & Filipps, 2024).

The third dimension, training in intercultural mediation and diversity, reflects the developmental component of intercultural competence. Contemporary research has highlighted the importance of formal and continuous training processes in fostering intercultural knowledge, mediation strategies, and professional preparedness for managing culturally complex situations (Perlinger & Wischmann, 2025; Dai & Feng, 2025). In law enforcement contexts, such training has been associated with improved professional effectiveness, institutional legitimacy, and cooperation with culturally diverse communities (Dai & Feng, 2025).

Consequently, the questionnaire was designed around three theoretically differentiated but related dimensions: (a) acceptance of intercultural environments, (b) intercultural competences in the workplace, and (c) training in intercultural mediation and diversity. The subsequent exploratory and confirmatory factor analyses were conducted to examine whether the empirical structure of the instrument was consistent with this theoretically grounded model.

Although several instruments have been developed to assess intercultural competence in educational, organizational, and community settings, none of them were specifically designed to evaluate intercultural mediation among law enforcement personnel operating in border contexts. Existing measures primarily focus on general intercultural attitudes, personality traits, or communication skills, without addressing the specific professional demands associated with public security work in culturally diverse environments. The present instrument seeks to fill this gap by integrating attitudinal, professional, and training-related dimensions within a single measure adapted to the realities of border law enforcement agencies. Consequently, the questionnaire provides a context-specific assessment tool with potential applications in professional training, institutional evaluation, and evidence-based policy development.

Based on the theoretical framework and previous research on intercultural competence and psychometric scale development, the following hypotheses were formulated:

**H1.**  *The instrument will exhibit a multidimensional structure composed of three factors, will demonstrate an acceptable model fit confirmed through confirmatory factor analysis, will show adequate reliability (internal consistency and test–retest stability), and will provide acceptable evidence of construct validity, including convergent and discriminant validity*.

## 2. Materials and Methods

### 2.1. Development of the Instrument

In the first phase, a review of the relevant literature was conducted, along with an examination of instruments related to the study topic, such as the Post-Intercultural Communicative Competence (POST-ICC) (Alghasab & Alvarez-Ayure, 2023), adapted from the Intercultural Communicative Competence (ICC) model (Byram, 1997), the Intercultural Competence Measurement Questionnaire (Ayala-Asencio, 2020), the Multicultural Personality Questionnaire (MPQ) (Van der Zee & Van Oudenhoven, 2001; Hofhuis et al., 2020), the Intercultural Development Inventory (IDI) (Hammer et al., 2003; Moon et al., 2018), the Multifactor Leadership Questionnaire (MLQ) (Bass & Avolio, 1995), the Intercultural Readiness Check (IRC) (Van der Zee & Brinkmann, 2004; Brinkmann & van Weerdenburg, 2014), and the Multicultural Personality Questionnaire—Short Form (MPQ-SF) (Van der Zee et al., 2013).

Subsequently, the instrument was validated using expert judgment. The initial version of the scale consisted of 42 items: 17 items measuring acceptance of intercultural environments, 16 items assessing intercultural competencies in the workplace, and 9 items evaluating training in mediation and intercultural diversity. A panel of 13 experts participated in the evaluation process. Seven were specialists in intercultural mediation (including educators, healthcare professionals, and law enforcement personnel), while six had research expertise in the social inclusion of culturally diverse populations.

The instrument and evaluation guidelines were distributed via email. Experts were asked to assess the appropriateness, relevance, and clarity of each item using a 5-point Likert scale. The Content Validity Index (CVI) results obtained from the expert evaluation are presented in Table 1. Based on the experts’ assessments of appropriateness, relevance, and clarity, items not meeting the established content validity criteria were removed or revised, resulting in the final version of the questionnaire (Table 1).

The content validation process was conducted through expert judgment, with each item evaluated in terms of appropriateness, relevance, and clarity. The results revealed a high level of agreement among experts, as most items obtained CVI values equal to or higher than 0.92 across the three evaluated dimensions. In addition, mean scores were consistently above 5.50 on a six-point scale, indicating that the items were considered conceptually adequate and clearly formulated.

Three items (Items 1, 11, and 41) did not reach the minimum acceptable threshold (CVI < 0.70) and were therefore removed from the instrument. In contrast, Item 42 showed intermediate CVI values (0.76–0.80). Following the experts’ recommendations, this item was revised and merged with a conceptually similar item to improve clarity and reduce redundancy.

After this refinement process, the questionnaire was reduced from 42 to 38 items. The high level of agreement observed among experts, together with the elimination or revision of problematic items, provides strong evidence of content validity and supports the use of the final version of the instrument in subsequent psychometric analyses.

### 2.2. Scale Scoring

All categories of the instrument are measured using a Likert scale ranging from ‘never’ (1) to ‘always’ (5). Thus, participants’ level of acceptance of intercultural environments is assessed through sixteen items, yielding a possible score range of 16 to 80. Intercultural competencies in the workplace are measured by fourteen items, yielding a possible score range of 14 to 70. Training in mediation and intercultural diversity is measured by eight items, yielding a possible score range of 8 to 40.

### 2.3. Pilot Study

The study was conducted following the ethical recommendations proposed in previous research in the same field and observing the principles and recommendations of the Declaration of Helsinki (1975), subsequently updated in Brazil in 2013 and by the Ethics Committee of the University of Granada (reference code no.: 4836/CEIH/2025). The instrument was pilot-tested with a sample of 20 public employees from the State Security Forces stationed in Spanish border areas, whose characteristics were similar to those of the study’s target population. It is important to note that all participants provided informed consent. The objective of this preliminary phase was to assess the questionnaire’s readability, estimate the time required to complete it, and analyze the degree of understanding of the items. The results demonstrated adequate understanding of the questions by the participants, as well as the feasibility of the instrument’s application in terms of clarity and response time, which supported its suitability for use in the final sample.

### 2.4. Sample and Procedure

A total of 938 public employees working in law enforcement agencies in Spain’s border regions participated in the study. Participants were selected using snowball sampling, which allowed researchers to reach professionals in this field through initial contacts who facilitated the recruitment of new participants. Due to the snowball sampling procedure, the exact number of individuals who received the invitation to participate could not be determined. Therefore, a response rate could not be accurately calculated. The recruitment period took place from 19 February 2025 to 18 December 2025. The use of snowball sampling was considered appropriate due to the difficulty of accessing active law enforcement personnel, a population that is often restricted for research purposes due to institutional and operational constraints. This sampling strategy is commonly employed in studies involving security forces and other hard-to-reach professional groups, where formal access channels are limited and participation depends on trust-based networks. Therefore, its use in the present study is justified by the specific characteristics of the target population and the need to ensure an adequate sample size. Inclusion criteria were: (a) being an active law enforcement professional working in the Spanish border regions of Ceuta or Melilla, (b) being at least 18 years old, and (c) voluntarily agreeing to participate in the study. Questionnaires with incomplete responses were excluded from the analyses.

In the Exploratory Factor Analysis (EFA) phase, the sample consisted of 426 participants. 66.8% were men and 33.2% were women, with a mean age of 42 years (SD = 8.1). Regarding geographic distribution, 62.3% worked in Melilla and 37.7% in Ceuta. In the Confirmatory Factor Analysis (CFA) phase, 512 public employees participated. In this case, 73.3% worked in Melilla and 26.7% in Ceuta. Overall, after combining both samples, the geographic distribution was 68.2% in Melilla and 31.8% in Ceuta. The participants included in the EFA and CFA phases were fully independent, and no individual participated in both stages of the validation process. This confirmed the participant-to-item ratio (10:1) recommended in the literature (Regnoli et al., 2025).

To ensure institutional anonymity, participants were broadly grouped within border security forces and agencies, without distinguishing between specific units. The questionnaires were administered in paper and online formats, in public spaces, to those officers who were available. Throughout the data collection process, the confidentiality of the information and the anonymity of the participants were ensured (Table 2).

### 2.5. Reliability Testing

The reliability of the instrument was assessed using two complementary methods: test–retest reliability and internal consistency. To evaluate test–retest reliability, the questionnaire was administered twice to 40 participants, with a two-week interval between administrations. Results indicated good temporal stability, with an intraclass correlation coefficient of ICC = 0.82 (95% CI = 0.77–0.86) for single measures and ICC = 0.90 for average measures, as well as a strong Pearson correlation between both administrations (r = 0.84, *p* < 0.001) (García-García et al., 2025; Van Druten et al., 2026). For the internal consistency analysis, the questionnaire was administered to 426 public employees in Melilla and Ceuta, yielding excellent overall reliability (α = 0.928).

### 2.6. Construct Validity

To establish the construct validity of the instrument, two different approaches to factor analysis were used. The first was exploratory factor analysis (EFA), and the second was confirmatory factor analysis (CFA). Confirmatory factor analysis was conducted using structural equation modeling.

Following criteria widely accepted in the recent literature, factors with eigenvalues greater than or equal to 1.0 were retained, and only items with factor loadings equal to or greater than 0.30 were considered significant for each factor in the exploratory factor analysis. The varimax rotation method was applied to verify that the factors successfully measured whether the subjects possessed the social skills necessary for successful integration. These criteria have been applied in recent psychometric studies (Lawson et al., 2026).

For the CFA, model fit was evaluated using multiple fit indices, including χ^2^, χ^2^/df, CFI, TLI, and RMSEA. Following commonly accepted criteria, values of CFI and TLI close to or above 0.90 and RMSEA values below 0.08 were considered indicative of acceptable model fit.

The latest versions of the statistical software SPSS 28 and Amos 28 were used to analyze the data.

## 3. Results

### 3.1. Descriptive Statistics

Descriptive statistics and an analysis of the internal consistency of the Intercultural Mediation Questionnaire for Public Employees in Law Enforcement Agencies in Spanish Border Contexts (CMIEPFS). Table 3 shows the descriptive statistics for the factors comprising the questionnaire (CMIEPFS): mean, skewness, kurtosis, range of scores, and Cronbach’s alpha coefficient. The dimensions exhibited skewness and kurtosis values consistent with approximate normality.

The internal consistency coefficients ranged from 0.805 to 0.928, indicating that the instrument has an excellent level of reliability.

### 3.2. Reliability and Validity

The construct validity of the instrument was examined through exploratory factor analysis (EFA) and confirmatory factor analysis (CFA). This analysis was conducted using data from 426 participants who responded to the 38 items in the questionnaire. The statistical software SPSS version 31 was used. The Kaiser–Meyer–Olkin measure was 0.916 and Bartlett’s test of sphericity was significant (χ^2^ = 7341.065, df = 703, *p* < 0.001), supporting the suitability of the data for exploratory factor analysis (Table 4).

All items showed communalities above 0.30 (Table 5). The analyzed instrument meets this criterion for all items. The exploratory factor analysis grouped the 38 items into three factors. Rotated factor loadings ranged from 0.381 to 0.741, and the three-factor solution explained 63.08% of the total variance (Table 6). These three factors account for 63.08% of the total explained variance.

**Figure 1:**
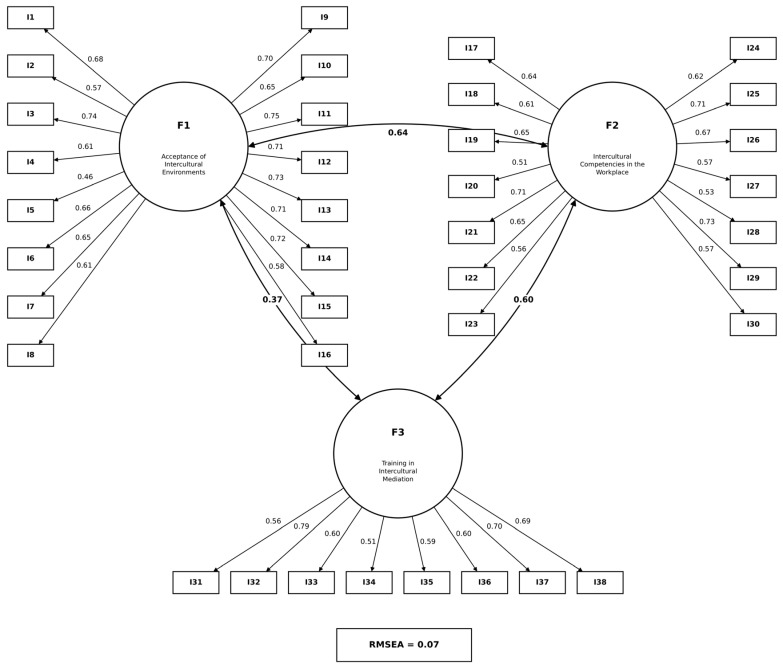
Confirmatory factor analysis model with standardized factor loadings. *Note.* Rectangles represent observed variables, circles represent latent factors, and double-headed arrows represent the covariances among the three latent factors: acceptance of intercultural environments (F1), intercultural competences in the workplace (F2), and training in intercultural mediation (F3).

Finally, a confirmatory factor analysis (CFA) was conducted to develop the final instrument (Figure 1). To perform this analysis, the questionnaire was administered again to public employees working in law enforcement agencies in Spanish border areas, in this case to a sample of 512 participants. Figure 1 presents the CFA model using standardized factor loadings. The complete set of standardized estimates is reported in Table 7.

The confirmatory factor analysis supported the proposed three-factor structure. The model showed an acceptable fit to the data (χ^2^ = 2711.472, df = 662, χ^2^/df = 4.096, CFI = 0.960, TLI = 0.931, RMSEA = 0.070). According to commonly accepted criteria, these values indicate an adequate fit between the proposed measurement model and the observed data. The standardized factor loadings are presented in Figure 1. These results support the adequacy of the proposed measurement model (Table 7).

### 3.3. Internal Consistency Analysis

The internal consistency of the questionnaire was reassessed using Cronbach’s alpha coefficient for this phase. The results showed high internal consistency for the set of 38 items, with a value of α = 0.91, indicating adequate homogeneity among the scale items. The questionnaire showed high internal consistency (α = 0.91).

### 3.4. Test–Retest Reliability

The test–retest reliability of the questionnaire was assessed using the intraclass correlation coefficient (ICC) via a two-factor mixed-model analysis with absolute agreement. The results showed an ICC of 0.82 for single measurements (95% CI = 0.77–0.86; *p* < 0.001), indicating good test–retest reliability of the instrument between the two administrations of the questionnaire. Likewise, the ICC for mean measurements was 0.90, suggesting excellent reliability when both measurements are considered together.

### 3.5. Correlation Between Measurements

In addition, Pearson’s correlation coefficient was calculated between the total scores obtained on the first and second administrations of the questionnaire. The correlation between administrations was strong and statistically significant (r = 0.84, *p* < 0.001) (Table 8).

Reliability analyses showed that the questionnaire exhibits adequate psychometric properties. Internal consistency was high, as evidenced by both Cronbach’s alpha (α = 0.91) and McDonald’s omega (ω = 0.92), indicating a high degree of homogeneity among the items and providing robust evidence of scale reliability. Furthermore, the test–retest reliability analysis revealed good temporal stability, with an intraclass correlation coefficient of 0.82 (95% CI = 0.77–0.86). Consistent with this finding, the correlation between the total scores obtained in both administrations was strong and statistically significant (r = 0.84, *p* < 0.001), confirming the stability of the instrument over time.

Consistent with these reliability results, the factor analysis also provided evidence of the instrument’s structural validity. Standardized factor loadings ranged from 0.43 to 0.73, indicating that all items contributed significantly to their respective latent constructs. Most loadings exceeded the recommended threshold of 0.50, supporting the adequate representation of the items. Standardized factor loadings ranged from 0.43 to 0.73, indicating that all items contributed to their respective latent constructs. Table 9 presents the standardized factor loadings and squared loadings for all questionnaire items. The standardized loadings correspond to those displayed graphically in Figure 1.

All standardized factor loadings reported in Table 9 correspond exactly to the values presented graphically in Figure 1; any minor discrepancies are due exclusively to rounding. Although all standardized factor loadings exceeded the minimum acceptable threshold (0.40), several items showed moderate loadings, particularly within the Training in Intercultural Mediation factor (F3). These results help explain the moderate AVE values observed for some factors and suggest that future refinements may focus on revising or replacing items with lower standardized loadings to strengthen convergent validity.

Convergent validity was assessed using Average Variance Extracted (AVE) and Composite Reliability (CR). The CR values ranged from 0.77 to 0.91, whereas AVE values ranged from 0.30 to 0.41 (Table 10).

Therefore, the results indicate that, although the proportion of variance explained by the latent constructs is moderate, the internal consistency and theoretical coherence of the factors support their adequacy. Nevertheless, future research should consider refining or eliminating items with lower factor loadings in order to improve the average variance extracted and strengthen the convergent validity of the instrument (Table 10).

Discriminant validity was assessed using the Fornell–Larcker criterion. The results provided partial support for discriminant validity. Although the square root of the Average Variance Extracted (AVE) exceeded most inter-factor correlations, the value for F2 was slightly lower than its correlation with F1, and the value for F3 was equivalent to its correlation with F2. These findings suggest that the three factors are conceptually related while remaining theoretically distinguishable dimensions of intercultural competence. The Fornell–Larcker criterion provided only partial support for discriminant validity. Although the factors remained theoretically distinguishable and most comparisons met the recommended threshold, some inter-factor correlations approached or slightly exceeded the corresponding square root of AVE values. Therefore, discriminant validity should be interpreted with caution and requires further examination in future studies (Table 11).

## 4. Discussion

The present study provides evidence regarding the validity and reliability of the instrument designed to assess intercultural competencies in professional contexts. By combining exploratory factor analysis (EFA) and confirmatory factor analysis (CFA), the study follows current best practices in psychometric validation, ensuring a rigorous evaluation of the latent structure underlying intercultural attitudes, knowledge, and skills (Boateng et al., 2018).

The results indicate that the data are highly suitable for factor analysis. The Kaiser–Meyer–Olkin measure (KMO = 0.916) reflects excellent sampling adequacy, exceeding the threshold of 0.90 recommended in recent methodological literature, while the significant Bartlett’s test of sphericity (χ^2^ = 7341.065, df = 703, *p* < 0.001) confirms the presence of sufficient correlations among items. These values are slightly higher than those reported in similar validation studies in intercultural competence, where KMO indices typically range between 0.80 and 0.90 (Beltrán-Sánchez et al., 2024), suggesting a particularly strong factorial structure in the present sample.

The EFA revealed factor loadings ranging from 0.516 to 0.741 for most items, which is consistent with previous research on complex psychosocial constructs, where moderate loadings are expected due to the multidimensional nature of the variables (Khademi et al., 2024; Genkova & Schreiber, 2025). Although one item presented a lower loading (0.381), its retention can be justified on theoretical grounds, as intercultural competence encompasses heterogeneous dimensions that are not always strongly correlated at the empirical level. This pattern aligns with findings from other intercultural competence scales, where variability in item loadings reflects the broad conceptual scope of the construct rather than measurement weakness (Chen & Gabrenya, 2021).

The three-factor structure identified—acceptance of intercultural environments, intercultural competences in the workplace, and training in mediation and intercultural diversity—is consistent with multidimensional models proposed in the literature (Gómez et al., 2023; Jin et al., 2026). However, the moderate correlations observed between factors suggest that, while empirically distinguishable, these dimensions are conceptually interrelated. This finding reinforces the idea that intercultural competence operates as an integrated system of attitudes, skills, and knowledge rather than as completely independent domains (Genkova & Schreiber, 2025).

From a theoretical perspective, these findings support contemporary models that conceptualize intercultural competence as an integrated system of attitudes, professional skills, and developmental experiences rather than as a single personality trait or isolated ability. The distinction between acceptance of intercultural environments, workplace competences, and intercultural mediation training reinforces the idea that effective intercultural performance emerges from the interaction between dispositional orientations, context-specific skills, and learning opportunities. This interpretation is consistent with other multidimensional frameworks (Deardorff & Jones, 2021; Gómez et al., 2023; Genkova & Schreiber, 2025).

The CFA results provide further support for the proposed model. The fit indices (CFI = 0.960, TLI = 0.931, RMSEA = 0.07) fall within the acceptable to excellent range for complex models and are comparable to those reported in similar validation studies (Andino-González et al., 2025; Sathyanarayana & Mohanasundaram, 2024). Although the chi-square statistic was significant, this result is expected given the large sample size and should not be interpreted in isolation, as widely acknowledged in structural equation modeling literature (Akbar et al., 2023; Chomeya & Tayraukham, 2025). Overall, the model demonstrates a strong capacity to represent the underlying data structure (Ikram et al., 2025; Qin et al., 2025).

Regarding reliability, the instrument shows high internal consistency (α = 0.91), exceeding the commonly accepted threshold of 0.80 and aligning with recent validation studies in social and behavioral sciences (Boateng et al., 2018). The test–retest reliability results (ICC = 0.82; r = 0.84) further confirm the temporal stability of the instrument, with values comparable to or higher than those reported in similar psychometric studies involving professional populations (Mia et al., 2025; Van Druten et al., 2026). These findings suggest that the instrument provides consistent and stable measurements over time, which is essential for its application in both research and professional evaluation contexts.

The practical implications of these findings are particularly relevant for law enforcement agencies operating in culturally diverse border regions. The instrument may be used to identify strengths and training needs among professionals who regularly interact with migrant, refugee, and culturally heterogeneous populations. In addition, the questionnaire may support the design of targeted professional development programs, facilitate the evaluation of intercultural training initiatives, and provide evidence for institutional decision-making processes aimed at improving service quality, conflict prevention, and community trust.

Despite these positive findings, several limitations should be acknowledged. First, convergent validity was only partially supported, as the AVE values ranged from 0.30 to 0.41, below the conventional threshold of 0.50. Although this limitation is partially compensated by the high composite reliability values, it suggests that the proportion of variance explained by the latent constructs is moderate. This pattern is not uncommon in the measurement of complex constructs such as intercultural competence, where conceptual breadth and item heterogeneity tend to reduce shared variance among indicators (Fornell & Larcker, 1981). Nevertheless, convergent validity can still be considered acceptable when composite reliability is sufficiently high (CR ≥ 0.70), as is the case in the present study. Future research should therefore focus on refining or replacing items with lower factor loadings in order to improve AVE values and strengthen the convergent validity of the instrument.

A further limitation concerns the presence of several items with moderate factor loadings. Although all items met the minimum retention criteria and contributed meaningfully to their respective constructs, some standardized loadings were moderate in magnitude. This result suggests that certain indicators may capture more specific or context-dependent aspects of intercultural mediation than others. While their retention was supported by theoretical considerations and by the overall adequacy of the model, future research should evaluate the performance of these items in different samples and professional contexts. Additional item refinement may contribute to improving factorial saturation and increasing the overall explanatory power of the instrument.

Another limitation of the present study is the use of snowball sampling. Although this strategy facilitated access to a professional population that is difficult to recruit through conventional procedures, it may limit the representativeness of the sample and the generalizability of the findings. Future studies should attempt to replicate the validation process using probabilistic or institutionally coordinated sampling procedures whenever feasible.

An additional limitation concerns the potential influence of social desirability bias. Given the professional context and the attitudinal nature of several items, participants may have been inclined to provide socially acceptable responses regarding intercultural attitudes and behaviors. Future studies should examine the relationship between questionnaire scores and external criteria such as supervisor evaluations, behavioral indicators, or alternative measures of intercultural competence.

An additional limitation of this study concerns the exclusive use of self-report measures. Although this approach is common in the assessment of intercultural competence, participants may provide responses that are influenced by social desirability, particularly in professional contexts such as law enforcement, where intercultural sensitivity and respect for diversity are institutionally valued. Consequently, some attitudes and self-perceived competences may have been overestimated. Future studies should complement self-report data with external sources of evidence, such as supervisor ratings, peer evaluations, behavioral indicators, or performance-based assessments, in order to further examine the criterion-related validity of the instrument and its correspondence with observable intercultural practices. An important limitation concerns discriminant validity. Although the three dimensions were theoretically distinct and showed acceptable reliability, the Fornell–Larcker criterion provided only partial support for discriminant validity. This finding suggests that the dimensions are closely related aspects of intercultural competence and that future studies should continue examining the distinctiveness of the factors using alternative approaches and independent samples.

Taken together, the convergence of evidence from factor analysis, reliability testing, and model fit indices supports the adequacy of the psychometric properties of the instrument, while also highlighting areas that require further refinement. The results reinforce the inherent complexity of measuring intercultural competence and the need for multidimensional and context-sensitive assessment approaches. Although the questionnaire was developed and validated within Spanish border law enforcement contexts, it shows potential for adaptation and future validation in other institutional settings characterized by cultural diversity, such as public administration, healthcare, and education.

However, the present findings should be interpreted within the specific context of law enforcement personnel working in the Spanish border regions of Ceuta and Melilla. Consequently, the applicability of the instrument to other professional sectors, geographical regions, or national contexts cannot be assumed and should be confirmed through additional validation studies.

In practical terms, the scale provides a useful tool for assessing intercultural competencies, facilitating the identification of training needs, and evaluating intervention programs. In line with previous research, the systematic assessment of intercultural competence can contribute to improving institutional effectiveness, promoting social cohesion, and strengthening trust between public institutions and culturally diverse populations (De Waal & Born, 2020).

## 5. Conclusions

This study has enabled the development and validation of a questionnaire to assess intercultural mediation among public employees in law enforcement agencies who work in Spanish border areas. The results provide evidence of adequate psychometric properties, including satisfactory construct validity and strong reliability indices. Although the findings support the usefulness of the instrument, some limitations—particularly the moderate AVE values and the need for further refinement of certain items—suggest that additional validation studies are warranted.

Exploratory and confirmatory factor analysis confirmed the existence of a three-dimensional structure comprising the dimensions of acceptance of intercultural environments, intercultural competencies in the workplace, and training in mediation and intercultural diversity. Furthermore, the internal consistency and test–retest reliability indices demonstrated the instrument’s high reliability, supporting the internal consistency of the items and the stability of the measurements over time.

Overall, the results indicate that the questionnaire is a valid and reliable tool for assessing competencies related to intercultural mediation in professional contexts characterized by high cultural diversity. Its use can contribute both to institutional assessment and to the design and evaluation of training programs aimed at strengthening the intercultural competencies of professionals working in multicultural settings.

Beyond the specific context of this study, the significance of these findings takes on an international dimension. In a global landscape marked by increasing migration flows, cultural diversity, and the growing complexity of interactions between institutions and citizens, the need for valid and comparable tools to assess intercultural competencies is essential. In this regard, the questionnaire developed has high potential for transfer and adaptation to other institutional and geographical contexts, including law enforcement agencies, public administrations, and organizations operating in multicultural environments across different countries.

In short, this study helps fill a gap in the literature regarding specific tools for assessing intercultural mediation in law enforcement agencies, providing a solid methodological foundation for future research and for the implementation of evidence-based training policies. From a scientific perspective, it offers a replicable tool that can facilitate international comparative studies and advance knowledge in the field of applied intercultural competence.

From a practical standpoint, the questionnaire helps identify the level of acceptance of intercultural environments, intercultural competencies in the workplace, and the extent of specific training in mediation and cultural diversity, thereby facilitating the assessment of training needs within institutions. Its use can contribute to the design of training programs better tailored to the real demands of multicultural contexts, improve the management of interactions with culturally diverse populations, and promote more effective, respectful, and conflict-prevention-oriented professional interventions, while simultaneously strengthening institutional trust and social cohesion in diverse contexts.

The relevance of the instrument is especially evident in Spanish border contexts such as Ceuta and Melilla, where law enforcement personnel frequently operate at the intersection of migration management, cultural diversity, and public security. In these environments, intercultural mediation competencies may contribute not only to more effective professional performance but also to the promotion of institutional legitimacy and constructive relationships between public authorities and culturally diverse communities. These findings should be interpreted in light of several limitations, including the use of non-probabilistic sampling, the exclusive focus on law enforcement personnel in Spanish border regions, and the moderate evidence of convergent validity observed in some dimensions. Future research should replicate the study in other professional and cultural contexts, examine measurement invariance, and continue refining the scale to strengthen its psychometric performance.

## 6. Limitations and Future Research Directions

Despite the study’s methodological soundness and the excellent reliability levels achieved, it is necessary to point out certain limitations that should be taken into account when interpreting the results.

First, although the sample size is relatively large (N = 938), the sampling strategy was based on non-probabilistic convenience sampling. Participants were recruited in accessible public settings, which may introduce selection bias and limit the representativeness of the sample. Consequently, the findings should be interpreted with caution, as they may not be fully generalizable to all law enforcement personnel or to other institutional or geographical contexts. Future research should aim to employ probabilistic sampling techniques or more controlled recruitment procedures to enhance the external validity and generalizability of the results. However, the relatively large sample size and the consistency of the results across independent subsamples partially mitigate this limitation. Another limitation concerns the convergent validity of the instrument. Although composite reliability values were satisfactory, the Average Variance Extracted (AVE) values were below the conventional threshold of 0.50. This indicates that the proportion of variance explained by the latent constructs is moderate, which may be attributed to the multidimensional and complex nature of intercultural competence, where heterogeneous indicators are expected. Similar patterns have been reported in previous validation studies of psychosocial constructs, where conceptual breadth can reduce shared variance among items. Future research should focus on refining the scale by reviewing or eliminating items with lower factor loadings, with the aim of improving the AVE values and strengthening the convergent validity of the instrument.

Therefore, future research should replicate the study in different regions and countries, as well as among other professional groups in the public sector—such as healthcare, education, or the judiciary—who also perform duties in intercultural settings. Such studies would allow for an evaluation of the instrument’s stability across different contexts and advance its cross-cultural validation.

Second, the cross-sectional design of the study limits the ability to establish causal relationships or to analyze changes in intercultural competencies over time. Although the test–retest analysis provides evidence of temporal stability, future research should incorporate longitudinal designs that allow for the assessment of the instrument’s sensitivity to changes resulting from specific training interventions, thereby reinforcing its usefulness as an impact assessment tool.

Third, the exclusive use of self-report measures may introduce biases associated with social desirability or the potential overestimation of professional competencies, especially in institutional contexts where certain attitudes may be perceived as normatively expected. In this regard, it would be advisable to supplement the questionnaire with multi-method approaches, such as 360° evaluations, behavioral observations, or qualitative methodologies, which allow for triangulation of information and improve the validity of the measurements.

Furthermore, although the instrument demonstrates excellent levels of internal consistency, it would be worthwhile to explore other psychometric indicators, such as convergent and discriminant validity, in relation to established scales of intercultural competence. Similarly, future studies should analyze factor invariance based on sociodemographic and professional variables (gender, professional group, years of experience) to ensure the model’s structural stability across different subgroups and contexts.

Finally, from an international perspective, it is particularly important to advance the cross-cultural adaptation of the instrument. Validation in different countries and institutional systems would not only reinforce its psychometric robustness but also facilitate comparative studies on intercultural competencies in diverse professional contexts, contributing to the development of global assessment frameworks in a context characterized by growing cultural diversity.

Taken together, these future lines of research will help strengthen the psychometric robustness of the instrument, optimize its factor structure, and expand its applicability across diverse intercultural contexts, thereby establishing it as a valid, reliable, and transferable tool for assessing and improving intercultural mediation in complex professional settings.

## 7. Practical Implications of the Study

The results of this study offer relevant practical implications for the management and training of public employees in law enforcement agencies operating in Spanish border contexts. The psychometric validation of the CMIEPFS questionnaire, with high internal consistency indices (overall α = 0.928) and a three-dimensional structure confirmed by EFA and CFA, provides a standardized and specific tool for assessing intercultural mediation in this professional group.

First, the instrument enables the institutional diagnostic assessment of three key dimensions: (1) acceptance of intercultural environments, (2) intercultural competencies in the workplace, and (3) training in mediation and intercultural diversity. This factor differentiation facilitates the identification of specific competency profiles within work teams, allowing for the detection of attitudinal strengths and potential training gaps, particularly in the dimension related to specific training in intercultural mediation.

Second, the questionnaire provides a solid empirical basis for the design, implementation, and evaluation of training programs tailored to the actual needs of the border context. Since the results show that the training dimension is a distinguishing factor, institutions can plan structured interventions—such as simulations of intercultural conflicts, training in non-criminal mediation strategies, or training in legal competencies related to the migrant population—aimed at strengthening practical skills that are directly transferable to professional performance.

Likewise, the tool can be used as an instrument for evaluating training impact, allowing for the measurement of pre-test–post-test changes following training programs, and for objectively assessing the effectiveness of internal diversity and inclusion policies.

This application is particularly relevant in contexts where institutional legitimacy and the proportionate management of cultural diversity influence the public’s perception of law enforcement agencies. From a strategic perspective, the systematic use of the questionnaire can contribute to the planning of evidence-based public policies aimed at preventing intercultural conflicts, improving coexistence, and strengthening public trust in complex multicultural contexts such as Ceuta and Melilla. The ability to obtain comparable data across different units, territories, or time periods facilitates decision-making based on objective indicators. Finally, the instrument appears suitable for assessing intercultural mediation competencies among law enforcement personnel in the Spanish border regions examined in this study. Nevertheless, further validation in other regions, professional groups, and cultural contexts is required before broader application can be recommended (Khademi et al., 2024; Andino-González et al., 2025).

## Figures and Tables

**Table 1:** Determining the Content Validity Index Through Expert Evaluation.

Item	Appropriateness Mean	SD	CVI	Relevance Mean	SD	CVI	Clarity Mean	SD	CVI
1	1.58	0.85	0.60	2.58	0.90	0.45	2.67	0.80	0.40
2	5.67	0.60	0.92	5.67	0.60	0.92	5.83	0.35	1.00
3	5.58	0.65	0.92	5.67	0.50	1.00	5.83	0.35	1.00
4	5.83	0.35	1.00	5.75	0.40	1.00	5.83	0.35	1.00
5	5.83	0.35	1.00	5.83	0.35	1.00	5.67	0.60	0.92
6	5.83	0.35	1.00	5.83	0.35	1.00	5.83	0.35	1.00
7	5.75	0.40	1.00	5.75	0.40	1.00	5.83	0.35	1.00
8	5.83	0.35	1.00	5.83	0.35	1.00	5.42	1.10	0.92
9	5.58	0.65	0.92	5.67	0.50	1.00	5.67	0.50	1.00
10	5.75	0.40	1.00	5.75	0.40	1.00	5.75	0.40	1.00
11	2.75	0.80	0.60	3.75	0.95	0.43	5.25	1.10	0.54
12	5.75	0.40	1.00	5.83	0.35	1.00	5.67	0.50	1.00
13	5.83	0.35	1.00	5.75	0.40	1.00	5.75	0.40	1.00
14	5.83	0.35	1.00	5.83	0.35	1.00	5.67	0.50	1.00
15	5.83	0.35	1.00	5.75	0.40	1.00	5.67	0.60	0.92
16	5.67	0.50	1.00	5.67	0.50	1.00	5.83	0.35	1.00
17	5.83	0.35	1.00	5.83	0.35	1.00	5.83	0.35	1.00
18	5.75	0.40	1.00	5.75	0.40	1.00	5.67	0.50	1.00
19	5.83	0.35	1.00	5.83	0.35	1.00	5.67	0.50	1.00
20	5.67	0.60	0.92	5.75	0.40	1.00	5.58	0.65	0.92
21	5.83	0.35	1.00	5.83	0.35	1.00	5.83	0.35	1.00
22	5.67	0.60	0.92	5.67	0.60	0.92	5.50	0.65	0.92
23	5.67	0.60	0.92	5.67	0.60	0.92	5.83	0.35	1.00
24	5.50	0.80	0.92	5.50	0.80	0.92	5.83	0.35	1.00
25	5.67	0.50	1.00	5.83	0.35	1.00	5.67	0.60	0.92
26	5.67	0.60	0.92	5.67	0.50	1.00	5.83	0.35	1.00
27	5.75	0.40	1.00	5.67	0.50	1.00	5.83	0.35	1.00
28	5.67	0.60	0.92	5.75	0.40	1.00	5.67	0.50	1.00
29	5.67	0.60	0.92	5.67	0.60	0.92	5.83	0.35	1.00
30	5.75	0.40	1.00	5.83	0.35	1.00	5.67	0.60	0.92
31	5.83	0.35	1.00	5.83	0.35	1.00	5.75	0.40	1.00
32	5.83	0.35	1.00	5.83	0.35	1.00	5.75	0.40	1.00
33	5.75	0.40	1.00	5.83	0.35	1.00	5.83	0.35	1.00
34	5.83	0.35	1.00	5.83	0.35	1.00	5.83	0.35	1.00
35	5.83	0.35	1.00	5.83	0.35	1.00	5.83	0.35	1.00
36	5.75	0.40	1.00	5.83	0.35	1.00	5.83	0.35	1.00
37	5.75	0.40	1.00	5.83	0.35	1.00	5.83	0.35	1.00
38	5.83	0.35	1.00	5.83	0.35	1.00	5.58	0.65	0.92
39	5.83	0.35	1.00	5.83	0.35	1.00	5.75	0.40	1.00
40	5.83	0.35	1.00	5.83	0.35	1.00	5.83	0.35	1.00
41	2.75	0.85	0.46	2.83	0.90	0.55	3.83	1.00	0.62
42	5.10	0.90	0.78	5.25	0.85	0.80	5.00	1.00	0.76

*Note.* Items 1, 11, and 41 were removed; Item 42 was revised and merged with a conceptually similar item, resulting in the final 38-item version.

**Table 2:** Distribution of participants across exploratory and confirmatory factor analysis samples by geographic location.

Province	EFA	CFA
Melilla	275	375
Ceuta	151	137
Total	426	512
Total Study	938	

**Table 3:** Descriptive statistics for the dimensions of the questionnaire (CMIEPFS).

Dimensions/Factors	Items	α	Mean	Skewness	Kurtosis	Range
F1	16	0.908	67.02	−0.41	1.36	87
F2	14	0.875	58.77	0.36	3.62	83
F3	8	0.805	25.01	1.72	7.83	82
Total	38	0.928				

*Note.* F1: acceptance of intercultural environments; F2: intercultural competencies in the workplace; F3: training in mediation and intercultural diversity; α: internal consistency coefficient (Cronbach’s alpha).

**Table 4:** Results of the Kaiser–Meyer–Olkin Measure and Bartlett’s Test of Sphericity for the Exploratory Factor Analysis.

Kaiser–Meyer–Olkin measure of sampling adequacy		0.92
Bartlett’s sphericity test	Approx. Chi-square	7341.065
	df	703
	*p*	0.000

**Table 5:** Item Communalities Following Exploratory Factor Analysis.

Items	Extraction
1. I believe that emotional intelligence is important for building meaningful relationships in intercultural contexts.	0.721
2. I respect the different ideas and opinions of other cultural, ethnic, or religious groups.	0.608
3. I view people from other cultures, ethnic groups, or religions as having equal value in society.	0.595
4. I believe that accepting people from different cultural/ethnic or religious backgrounds fosters acceptance of different opinions and realities.	0.687
5. I believe that society is strengthened by the recognition and acceptance of people from other cultures/ethnic groups or religions.	0.741
6. I believe that a society’s cultural diversity promotes positive intercultural interactions.	0.737
7. I believe that relationships with other cultures/ethnicities or religions require sensitivity, empathy, and tolerance.	0.611
8. I value interacting with people from other cultures/ethnicities or religions.	0.614
9. I demonstrate an open and unbiased attitude toward the different norms and values of other cultures/ethnicities and religions.	0.606
10. I engage with the traditions of other cultures/ethnic groups and religions.	0.599
11. I find environments characterized by cultural diversity interesting.	0.712
12. When faced with racist situations, I take the initiative to act against them.	0.569
13. I tend to feel comfortable in intercultural contexts and do not view them as a threat.	0.637
14. I enjoy interacting with people who have religious and/or cultural beliefs, as well as customs, that differ from my own.	0.619
15. I am able to establish and maintain friendly relationships with people from different cultural, ethnic, or religious groups.	0.576
16. A culturally diverse society does NOT cause stress for the members of the community involved.	0.516
17. In my workplace, I promote social inclusion for people from other cultures, ethnic groups, or religions.	0.620
18. I believe that the cultural diversity of the city where I work enriches my professional knowledge.	0.705
19. I strive to be empathetic or friendly toward the culturally diverse people I serve in my work.	0.516
20. I have sufficient knowledge of the cultural, ethnic, and religious context in which I perform my professional duties.	0.634
21. Through interaction with people from other cultures, ethnicities, or religions, I develop new positive perspectives that benefit my work.	0.731
22. I have sufficient skills to adapt to different work contexts shaped by diverse cultural, ethnic, or religious realities.	0.631
23. I believe that intercultural contexts enhance the professional development of public employees.	0.681
24. I avoid racist behavior to improve the intercultural relationships I encounter at work.	0.672
25. I understand the feelings and customs of people from different cultures/ethnicities or religions whom I encounter in my professional work.	0.646
26. I handle the various workplace situations that may arise as effectively as possible, regardless of the cultures, ethnicities, or religions of those involved.	0.700
27. I am able to empathize with the feelings, thoughts, and behaviors of people from other cultures, ethnicities, and religions whom I encounter in my professional work.	0.689
28. My beliefs have a positive influence on my professional conduct toward intercultural groups.	0.609
29. I avoid making judgments based on cultural, ethnic, or religious stereotypes in my workplace.	0.585
30. I am able to apply intercultural mediation strategies when dealing with culturally diverse individuals in my workplace.	0.720
31. In my workplace, there are training opportunities (courses, workshops, seminars) to expand my knowledge of intercultural diversity.	0.533
32. My training as a new civil servant included learning about intercultural issues.	0.611
33. I have received training in psychological and social aspects that foster understanding, empathy, and interaction with people of different ethnicities, cultures, or religions.	0.481
34. I am interested in and consider training through activities that simulate real intercultural conflicts, as well as activities that demonstrate the best ways to mediate them, to be essential for my work.	0.727
35. I have received training in other languages to improve my interaction with people from other countries	0.588
36. I have expanded my training in legal matters to understand the specific rights and responsibilities of immigrants	0.671
37. I have received training in mediation strategies that resolve intercultural conflicts (not crimes).	0.721
38. I consider training in intercultural issues relevant to my professional development	0.729

**Table 6:** Rotated Factor Loadings for the Three-Factor Structure of the CMIEPFS Questionnaire.

Items	Factors		
	1	2	3
1. I believe that emotional intelligence is important for building meaningful relationships in intercultural contexts.	0.563	0.228	0.130
2. I respect the different ideas and opinions of other cultural, ethnic, or religious groups.	0.563	0.061	0.188
3. I view people from other cultures, ethnic groups, or religions as having equal value in society.	0.466	0.175	0.003
4. I believe that accepting people from different cultural/ethnic or religious backgrounds fosters acceptance of different opinions and realities.	0.666	0.000	0.089
5. I believe that society is strengthened by the recognition and acceptance of people from other cultures/ethnic groups or religions.	0.669	0.245	0.186
6. I believe that a society’s cultural diversity promotes positive intercultural interactions.	0.733	0.214	0.013
7. I believe that relationships with other cultures/ethnicities or religions require sensitivity, empathy, and tolerance.	0.656	0.341	0.386
8. I value interacting with people from other cultures/ethnicities or religions.	0.694	0.054	0.324
9. I demonstrate an open and unbiased attitude toward the different norms and values of other cultures/ethnicities and religions.	0.674	0.345	0.092
10. I engage with the traditions of other cultures/ethnic groups and religions.	0.605	0.155	0.224
11. I find environments characterized by cultural diversity interesting.	0.721	0.351	0.420
12. When faced with racist situations, I take the initiative to act against them.	0.463	0.239	0.324
13. I tend to feel comfortable in intercultural contexts and do not view them as a threat.	0.679	0.229	0.332
14. I enjoy interacting with people who have religious and/or cultural beliefs, as well as customs, that differ from my own.	0.712	0.294	0.286
15. I am able to establish and maintain friendly relationships with people from different cultural, ethnic, or religious groups.	0.678	0.213	0.393
16. A culturally diverse society does NOT cause stress for the members of the community involved.	0.567	0.289	0.326
17. In my workplace, I promote social inclusion for people from other cultures, ethnic groups, or religions.	0.294	0.608	0.321
18. I believe that the cultural diversity of the city where I work enriches my professional knowledge.	0.089	0.601	0.217
19. I strive to be empathetic or friendly toward the culturally diverse people I serve in my work.	0.250	0.539	0.353
20. I have sufficient knowledge of the cultural, ethnic, and religious context in which I perform my professional duties.	0.289	0.519	0.364
21. Through interaction with people from other cultures, ethnicities, or religions, I develop new positive perspectives that benefit my work.	0.167	0.675	0.254
22. I have sufficient skills to adapt to different work contexts shaped by diverse cultural, ethnic, or religious realities.	0.240	0.581	0.172
23. I believe that intercultural contexts enhance the professional development of public employees.	0.377	0.702	0.046
24. I avoid racist behavior to improve the intercultural relationships I encounter at work.	0.249	0.528	0.205
25. I understand the feelings and customs of people from different cultures/ethnicities or religions whom I encounter in my professional work.	0.143	0.678	0.312
26. I handle the various workplace situations that may arise as effectively as possible, regardless of the cultures, ethnicities, or religions of those involved.	0.333	0.532	0.104
27. I am able to empathize with the feelings, thoughts, and behaviors of people from other cultures, ethnicities, and religions whom I encounter in my professional work.	0.170	0.628	0.291
28. My beliefs have a positive influence on my professional conduct toward intercultural groups.	0.259	0.525	0.169
29. I avoid making judgments based on cultural, ethnic, or religious stereotypes in my workplace.	0.357	0.480	0.222
30. I am able to apply intercultural mediation strategies when dealing with culturally diverse individuals in my workplace.	0.182	0.329	0.117
31. In my workplace, there are training opportunities (courses, workshops, seminars) to expand my knowledge of intercultural diversity.	0.210	0.172	0.547
32. My training as a new civil servant included learning about intercultural issues.	0.027	0.338	0.603
33. I have received training in psychological and social aspects that foster understanding, empathy, and interaction with people of different ethnicities, cultures, or religions.	0.230	0.296	0.525
34. I am interested in and consider training through activities that simulate real intercultural conflicts, as well as activities that demonstrate the best ways to mediate them, to be essential for my work.	0.123	0.135	0.490
35. I have received training in other languages to improve my interaction with people from other countries	0.230	0.123	0.425
36. I have expanded my training in legal matters to understand the specific rights and responsibilities of immigrants	0.187	0.107	0.563
37. I have received training in mediation strategies that resolve intercultural conflicts (not crimes).	0.060	0.243	0.662
38. I consider training in intercultural issues relevant to my professional development	0.312	0.132	0.547

**Table 7:** Confirmatory factor analysis fit indices for the proposed three-factor model.

Fit Index	Value
χ^2^	2711.472
df	662
*p*-value	<0.001
χ^2^/df	4.096
CFI	0.960
TLI	0.931
RMSEA	0.070
RMSEA 90% CI	[0.066, 0.074]
SRMR	0.076

**Table 8:** Reliability Indicators of the CMIEPFS Questionnaire.

Analysis	Result	Interpretation
Cronbach’s Alpha (α)	0.91	High internal consistency
McDonald’s omega (ω)	0.92	High internal consistency
ICC (single measures)	0.82	Good test–retest reliability
ICC (average measures)	0.90	Excellent reliability
Pearson correlation between T1 and T2	0.84	Strong relationship

**Table 9:** Standardized factor loadings and squared loadings.

Factor	Item	λ	λ^2^
F1—Acceptance of intercultural environments	Item 1	0.69	0.48
	Item 2	0.56	0.31
	Item 3	0.47	0.22
	Item 4	0.67	0.45
	Item 5	0.67	0.45
	Item 6	0.73	0.53
	Item 7	0.65	0.42
	Item 8	0.69	0.48
	Item 9	0.67	0.45
	Item 10	0.61	0.37
	Item 11	0.72	0.52
	Item 12	0.46	0.21
	Item 13	0.68	0.46
	Item 14	0.71	0.50
	Item 15	0.68	0.46
	Item 16	0.57	0.32
F2—Intercultural competences in the workplace	Item 17	0.61	0.37
	Item 18	0.60	0.36
	Item 19	0.54	0.29
	Item 20	0.52	0.27
	Item 21	0.68	0.46
	Item 22	0.58	0.34
	Item 23	0.70	0.49
	Item 24	0.53	0.28
	Item 25	0.68	0.46
	Item 26	0.53	0.28
	Item 27	0.63	0.40
	Item 28	0.52	0.27
	Item 29	0.48	0.23
	Item 30	0.49	0.24
F3—Training in intercultural mediation	Item 31	0.55	0.30
	Item 32	0.60	0.36
	Item 33	0.52	0.27
	Item 34	0.49	0.24
	Item 35	0.43	0.18
	Item 36	0.56	0.31
	Item 37	0.66	0.44
	Item 38	0.55	0.30

*Note.* Standardized factor loadings reported in this table correspond to the standardized estimates displayed in Figure 1. Minor differences are due to rounding.

**Table 10:** Convergent validity and composite reliability.

Factor	AVE	CR	ω
F1—Acceptance	0.41	0.91	0.92
F2—Competences	0.35	0.87	0.90
F3—Training	0.30	0.77	0.82

**Table 11:** Discriminant validity (Fornell–Larcker criterion).

Factor	√ (F1)	√ (F2)	√ (F3)
F1: Acceptance of intercultural environments	**0.64**	0.60	0.50
F2: Intercultural competences in the workplace	0.60	**0.59**	0.55
F3: Training in intercultural mediation	0.50	0.55	**0.55**

*Note.* Bold diagonal values = square root of the Average Variance Extracted (√AVE) for each factor. Off-diagonal values = inter-factor correlations. According to the Fornell–Larcker criterion, discriminant validity is supported when the square root of AVE exceeds inter-factor correlations.

## Data Availability

The datasets generated and analyzed during the current study are available from the corresponding author upon reasonable request.
